# A Rare Case of Asystole Due to Exaggerated Vagal Tone Following Chest Tube Placement

**DOI:** 10.7759/cureus.98599

**Published:** 2025-12-06

**Authors:** Muhammad Asif, Sukhila Reddy, Jurgen Shtembari, Talha Shamshad, Muhammad U Rana, Randolph Martin

**Affiliations:** 1 Cardiology, Carle Foundation Hospital, Urbana, USA; 2 Internal Medicine, Carle Foundation Hospital, Urbana, USA

**Keywords:** chest tube complication, chest tube management, increased vagal tone, reflex asystolic syncope, reversible bradycardia, transient asystole

## Abstract

Excessive vagal tone can lead to unstable bradyarrhythmias. We present the case of a 39-year-old otherwise healthy man who failed outpatient treatment for community-acquired pneumonia with oral antibiotics and underwent left-sided chest tube placement for empyema. After chest tube placement, the patient had a first-ever episode of syncope, which on telemetry coincided with a 16-second pause and asystole followed by the spontaneous recovery of sinus rhythm. Electrolytes and high-sensitivity troponin were within normal limits. The electrocardiogram (EKG) did not reveal any signs of ischemia. Eventually, the chest tube was removed after the drainage of pleural fluid, and the patient did not have any recurrence of arrhythmia or symptoms. He was discharged home after the completion of intravenous antibiotics.

## Introduction

Empyema is a well-known complication of pneumonia (PNA). The treatment of empyema requires the placement of a chest tube in addition to appropriate antibiotics. Chest tube placement is usually a safe procedure. However, it can rarely cause severe brady- or tachyarrhythmias [1]. Appropriate management requires clinicians' awareness of this rare complication to assist in prompt identification and guide treatment. Symptomatic sinus pauses secondary to chest tube insertion have been reported previously in the literature [2]. We report a rare case in which the sinus pause was unusually prolonged, lasting 16 seconds, leading to asystole recorded on telemetry and clinical syncope before the spontaneous return of sinus rhythm.

## Case presentation

**Figure 1:**
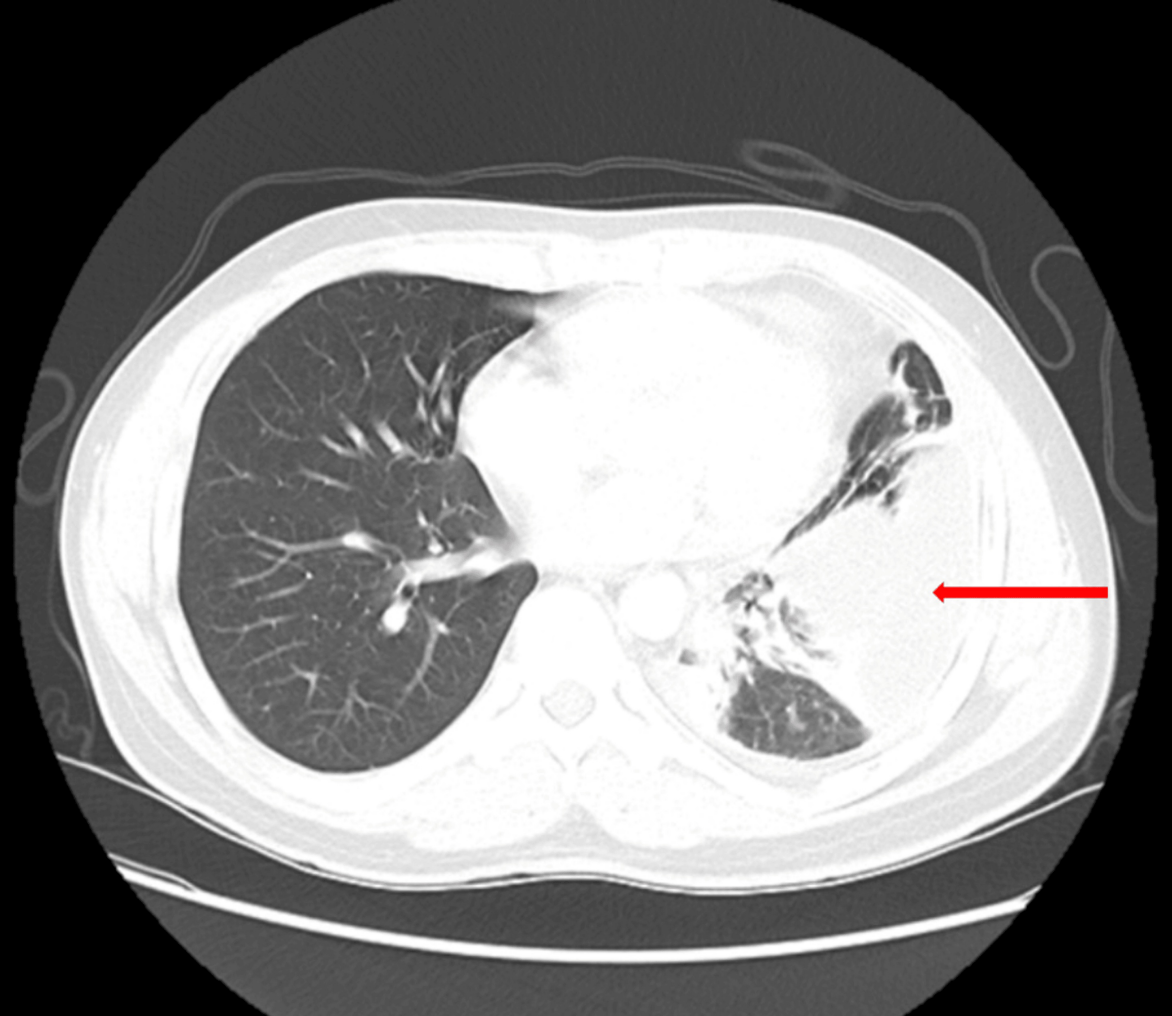
CT of the chest with contrast Red arrow indicating the loculated left-sided pleural effusion/empyema. CT: computed tomography

A 39-year-old man without any previous cardiac disease history presented with a non-productive cough, fever, and chills for 10 days. A review of systems was significant for pleuritic chest pain. Prior to arrival at the emergency room (ER), he was diagnosed with community-acquired pneumonia (CAP) as an outpatient for which he received five days of oral levofloxacin and two days of intravenous ceftriaxone without relief of symptoms. The patient then presented to the ER for further evaluation. In the ER, his vital signs were within normal limits, and his physical exam was consistent with decreased breath sounds at the left mid to lower lung field with rhonchi. Labs were significant for normal hemoglobin and neutrophilic leukocytosis with a white blood cell (WBC) count of 17×10^3^/uL (4-11×10^3^/uL) and a platelet count of 594×10^3^/uL (140-400×10^3^/uL). High-sensitivity troponin, lactic acid, and baseline electrocardiogram (EKG) were within normal limits. A computed tomography (CT) scan of the chest with contrast showed mixed airspace and interstitial infiltrate in the left lower lobe with a moderate-sized loculated pleural fluid collection suggestive of empyema (Figure 1).

The patient was started on IV vancomycin and piperacillin-tazobactam and was admitted to the hospital. He underwent left-sided chest tube placement by interventional radiology. To facilitate the drainage of the thick pleural fluid, he was started on tissue plasminogen activator (TPA)/dornase through the chest tube with intermittent suction. Soon after the first drainage of 120 ml fluid from the pleural space, the patient developed syncope, and the telemetry alarm went off with bradycardia, followed by sinus pause and asystole lasting for 16 seconds. By the time the patient was checked on in less than 30 seconds, the rhythm had returned to normal sinus rhythm (Video 1).

**Video 1:** Telemetry video showing the sinus rhythm changing to progressive bradycardia, asystole, and then spontaneous return of sinus rhythm

**Figure 2:**
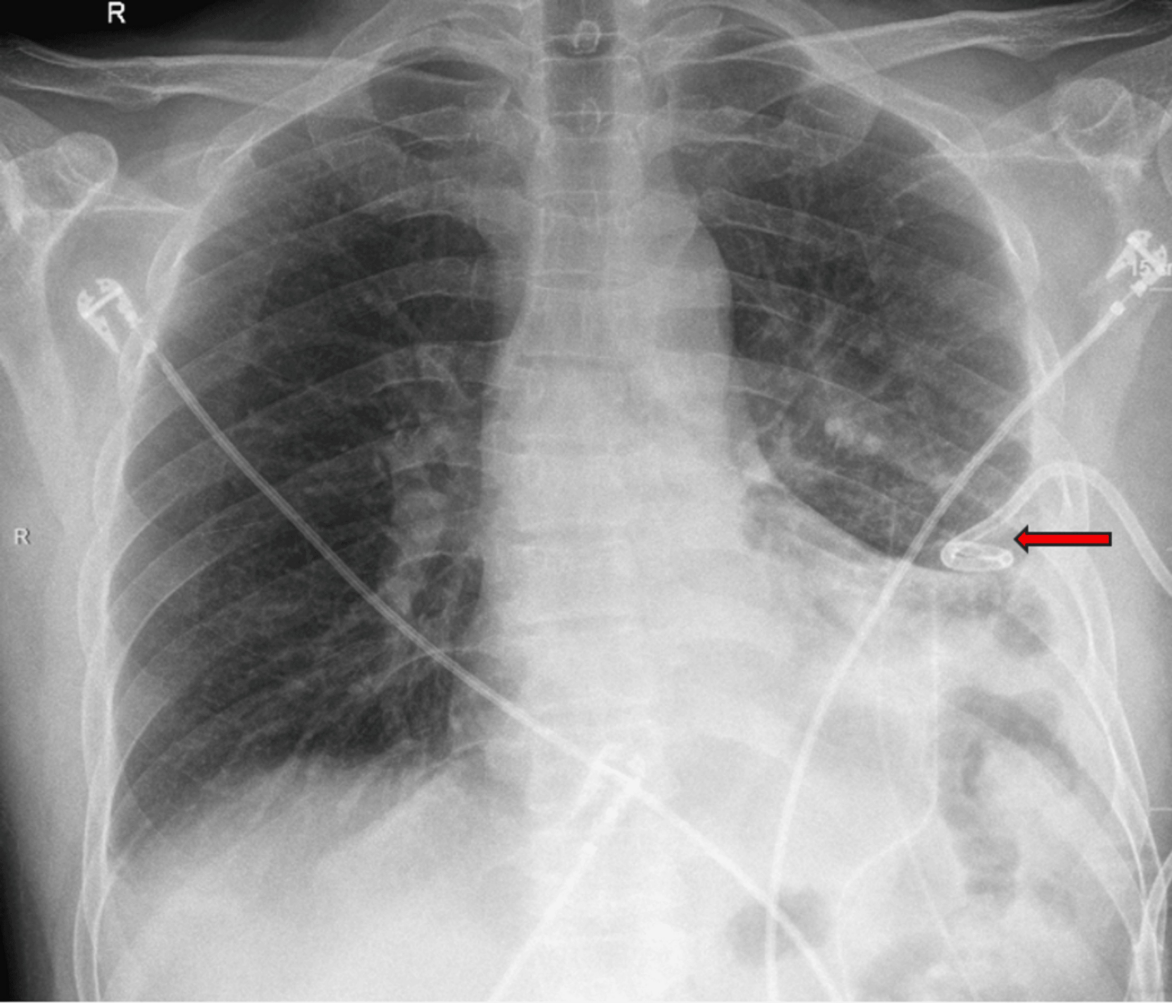
X-ray of the chest Red arrow indicating the left-sided infiltrate, pleural effusion, and chest tube in place.

The patient described being awake before the event, feeling "odd" for a few seconds, and then experiencing "darkness", and the next thing he remembered was seeing the code team around him. He felt fatigued for about five minutes before returning to baseline without any interventions. His vital signs were noted to be stable: blood pressure (BP) 106/61 mmHg, heart rate (HR) 65 beats/min, temperature 98.1°F, respiratory rate (RR) 22/min, and SpO2 96% on room air. Stat EKG, electrolytes, high-sensitivity troponin, and thyroid-stimulating hormone (TSH) were within normal limits. The follow-up chest X-ray was negative for any pneumothorax or chest tube displacement (Figure 2).

The case was discussed with cardiology, and the patient was monitored in the hospital for several days with telemonitoring during his inpatient stay, without any recurrence of symptoms or arrhythmias on telemetry. He denied having any family history of arrhythmias or prior syncope. The event was attributed to excessive vagal tone secondary to the intrathoracic pressure changes, pain caused by the chest tube, and underlying effusion. The chest tube was eventually removed following the resolution of the pleural effusion. The patient completed IV antibiotics and was discharged home on oral antibiotics for outpatient follow-up.

## Discussion

Reflex syncope is the most common cause of syncope in young patients without underlying structural heart disease. The vagal reflex has a significant role in cardiovascular regulation. The parasympathetic nervous system, through the vagus nerve, exerts a profound influence on the heart and is responsible for the autoregulation of cardiovascular function, including heart rate, rhythm, and blood pressure [3]. Minor vasovagal reactions have been previously reported in up to 16% of patients in one study after colonic insufflation during colonoscopy, due to the stimulation of gastrointestinal (GI) vagal receptors [4]. Sudden emotional stress, painful stimuli, changes in intrathoracic pressure, activation of vagal receptors in the thoracic cavity by stretch, or other noxious stimuli can trigger the vagal inhibition of the sinoatrial (SA) and atrioventricular (AV) nodes, leading to bradycardia, sinus pause, and, rarely, asystole [5]. On the other hand, excessive vagal stimulation also results in transient vasodilation through baroreceptors. A sudden decrease in heart rate and peripheral resistance leads to a drop in cerebral perfusion, resulting in syncope. The condition is mostly benign; however, if it persists long enough, it could result in cardiac arrest and be fatal [6].

A chest tube can increase vagal tone by various means, including pain from the puncture site, emotional distress due to fear of the procedure, direct nerve stimulation from the receptors in the pleural space, and sudden changes in intrathoracic pressure from underlying pneumothorax or hydrothorax. The event is more likely to occur during chest tube placement; however, it can occur throughout the chest tube stay, as its position may change with changes in body posture or with decreased volume of the pneumothorax or drainage fluid [2]. Our case not only verifies the previously reported findings in the literature, indicating that hemodynamically significant arrhythmias can be caused by excessive vagal tone, but also adds to them by being unique in that it was recorded on telemetry with a significantly long duration of asystole [2,4,5].

For chest tube placement, clinical status and vitals should be closely monitored. If bradycardia is noted during the chest tube insertion procedure, the procedure should be halted, and the patient should be stabilized before proceeding further. Post-procedurally, if severe vagal activation results in advanced bradycardia, standard Advanced Cardiovascular Life Support (ACLS) protocol, including atropine and as-needed chronotropic agents or pacing, should be considered in addition to reassessing the chest tube position and addressing the underlying etiology of bradycardia or asystole.Supposing there is no previous history of arrhythmia in the patient or family and no structural heart disease is suspected, in that case, these patients usually have a benign course after the underlying cause of increased vagal activation has been removed. A thorough history and clinical assessment of risk factors should be done for any baseline conduction disease, ischemia, or other etiology of syncope. Consultation with cardiology may also be considered to guide further surveillance or diagnostics [7].

## Conclusions

Activation of the parasympathetic system, triggered by severe pain or irritation of afferent visceral vagal receptors, can lead to excessive vagal tone and cardiovascular effects. The inhibitory effect of the vagus nerve on the SA node and AV node is mostly mild, resulting in asymptomatic bradycardia, sinus pause, or AV block; however, in extreme cases, it can be prolonged and symptomatic, leading to asystole. Adequate understanding of potential procedural complications and close post-procedural monitoring is crucial to timely diagnosis and management.
